# Editorial: Astrocytes and immunity: unveiling their role in pain and neurodegenerative disease progression

**DOI:** 10.3389/fnagi.2026.1944416

**Published:** 2026-08-11

**Authors:** Alessandra Toti, Roberta Facchinetti, Sumonto Mitra, Giulia Magni

**Affiliations:** 1 Department of Neuroscience, Psychology, Drug Research and Child Health-NEUROFARBA-Pharmacology and Toxicology Section, University of Florence, Florence, Italy; 2 Department of Life Sciences, Health and Health Professions, Link Campus University, Rome, Italy; 3 Department of Neurobiology, Care Sciences and Society, Division of Clinical Geriatrics, Center for Alzheimer Research, Karolinska Institutet, Huddinge, Sweden; 4 Neurology Unit, Department of Neurosciences, Fondazione IRCCS Ca' Granda Ospedale Maggiore Policlinico, Milan, Italy

**Keywords:** astrocytes, blood-brain barrier, mast cells, neurodegenerative diseases, neuroinflammation

Astrocytes are now recognized as active regulators of central nervous system homeostasis and disease. Through their interactions with neurons, immune cells, endothelial cells, and peripheral signals, they shape neurotransmission, inflammatory responses, ionic balance, and blood–brain barrier (BBB) integrity. The articles collected in this Research Topic illustrate this functional breadth and reinforce a central concept: astrocyte responses are highly context-dependent. They vary across anatomical regions, disease stages, biological sex, and pathological stimuli, and may shift from adaptive to maladaptive functions over time.

Two original studies address astrocyte-dependent mechanisms of pain sensitization. Liu et al. investigated chronic post-thoracotomy pain and identified astrocytic receptor tyrosine kinase-like orphan receptor 2 (Ror2) as a link between central neuroinflammatory changes and altered gut homeostasis. In male rats, chronic pain was associated with increased astrocytic Ror2 in the anterior cingulate cortex, a reactive profile enriched in A1-associated markers, enhanced CCL2 and CXCL1 expression, and reduced gut-derived short-chain fatty acids. Astrocyte-targeted Ror2 knockdown alleviated mechanical and cold hypersensitivity while partially reversing these central and peripheral alterations. Beyond identifying a candidate therapeutic pathway, this study suggests that astrocytic signaling may participate in distributed brain–gut responses to persistent pain. It also underscores the need to move beyond a rigid A1/A2 classification toward molecularly and functionally defined reactive states.

Patil et al. reveal a distinct astrocyte-mediated pathway in postoperative and inflammatory pain. Their findings identify extra-pituitary prolactin produced by spinal GLAST-positive astrocytes as an important contributor to female-selective hypersensitivity. Pharmacological prolactin receptor antagonism reduced nociceptive signaling and postoperative hypersensitivity in females, whereas ablation of GLAST-positive cells attenuated pain behavior and occluded the antagonist's additional effect. These observations position astrocytes as a local source of sex-dependent neuromodulatory signals and emphasize that biological sex should be treated as a mechanistic variable rather than merely a demographic characteristic in pain research.

The remaining contributions examine astrocyte dysfunction across neurodegenerative conditions. Huang et al. provide a bibliometric map of research on astrocytes and cognitive impairment from 2015 to 2024. Their analysis documents sustained growth in the field and identifies neuroinflammation as a persistent organizing theme, with synaptic dysfunction and single-cell technologies emerging as prominent research frontiers. Alzheimer's disease, Parkinson's disease, vascular dementia, traumatic brain injury, and the hippocampus dominate the literature. This overview highlights both the expanding recognition of astrocyte heterogeneity and the continuing need to translate mechanistic discoveries into clinically informative biomarkers and interventions.

Focusing on Parkinson's disease, Stoll and Harms synthesize evidence for a bidirectional astrocyte–vascular axis. Human imaging, fluid biomarkers, postmortem studies, and experimental models collectively indicate that astrocytic abnormalities and BBB dysfunction can coexist in vulnerable regions, including the substantia nigra and striatum. Altered end-foot polarization, inflammatory and angiogenic signaling, tight-junction disruption, and vascular remodeling may amplify neurodegeneration. However, the evidence remains heterogeneous, and the temporal relationship between barrier failure, astrocyte dysfunction, immune activation, and neuronal loss is unresolved. Longitudinal human studies and multicellular models of the neurovascular unit will therefore be essential for distinguishing causal drivers from secondary responses.

Samokhina and Buskila complement this neurovascular perspective by examining astrocytic potassium regulation in Alzheimer's disease and amyotrophic lateral sclerosis. Impaired Kir_4.1_-dependent potassium clearance, disrupted gap-junction coupling, and altered connexin-43 function may raise extracellular potassium, in turn promoting neuronal hyperexcitability, glutamate release, and excitotoxic vulnerability. Importantly, these changes appear region- and stage-specific and interact with mitochondrial dysfunction, oxidative stress, and inflammatory signaling. Astrocytic ionic homeostasis thus emerges not as a passive background process, but as a potential amplifier of selective neuronal vulnerability.

Together, these contributions show that astrocytes integrate signals across multiple biological scales—from membrane channels and chemokines to neural circuits, the neurovascular unit, and the gut–brain axis. They also expose shared challenges. Astrocyte states cannot be adequately captured by binary classifications; causality and temporal sequence remain difficult to establish; and findings from animal models require validation in human systems. Future work should integrate biological sex, aging and disease stage, single-cell and spatial profiling, electrophysiology, longitudinal imaging, and patient-derived multicellular models.

Therapeutically, the studies in this Research Topic support targeting specific dysfunctional astrocytic pathways rather than globally suppressing astrocyte reactivity. Ror2 signaling, local prolactin signaling, Kir_4.1_ and connexin function, AQP4 localization, and astrocyte–endothelial communication represents promising examples. The central task will be to identify when these pathways are protective, compensatory, or pathogenic. By defining this context, astrocyte-centered strategies may contribute to more precise treatments for chronic pain and neurodegenerative disease.

